# *JAK2* Inhibition: Reviewing a New Therapeutical Option in Myeloproliferative Neoplasms

**DOI:** 10.1155/2012/535709

**Published:** 2012-02-16

**Authors:** Mar Bellido, Peter A. W. te Boekhorst

**Affiliations:** Department of Hematology, Erasmus University Medical Center, P.O. Box 2040, 3000 CA Rotterdam, The Netherlands

## Abstract

*JAK2* is a tyrosine kinase gene that plays an essential role in the development of normal haematopoiesis. Hyperactivation of *JAK2* occurs in myeloproliferative neoplasms by different mechanisms. As a consequence, *JAK2* inhibitors have been designed to suppress the cytokine signalling cascade caused by the constitutive activation of *JAK2*. In clinical trials, *JAK2* inhibitors are efficient in decreasing spleen size, controlling clinical symptoms, and improving quality of life in patients with myeloproliferative neoplasms. However, *JAK2* inhibitors are unable to target uncommitted hematopoietic progenitors responsible of the initiation of the myeloproliferative disease. It is expected that, in order to cure the myeloproliferative disease, *JAK2* inhibitors should be combined with other drugs to target simultaneously different pathways and to target the initiator hematopoietic cell population in myeloproliferative disorders. Taking advantage of the inhibition of the cytokine cascade of *JAK2* inhibitors, these compounds are going to be used not only to treat patients with hematological neoplasms but may also be beneficial to treat patients with rheumatoid arthritis or other inflammatory diseases.

## 1. Introduction

Myeloproliferative neoplasms (MPNs) are clonal disorders up to now characterized by the autonomous proliferation of committed hematopoietic progenitors secondary to an aberrant activation of tyrosine kinase (TK) signalling pathways in combination with an exaggerated response to hematopoietic cytokines and growth factors [1, 2]. Constitutive activation of TKs is a consistent molecular signature in cell proliferation. Examples of Constitutive activation of TKs are seeing in solid tumours [3, 4], rheumatoid arthritis [5], and hematopoietic malignancies [6]. Known mechanisms of TK activation may result from acquired heterozygote of homozygote point mutations [7, 8], internal tandem duplications [9], and chromosomal translocations [10, 11]. The knowledge of the molecular mechanism involved in the pathogenesis of chronic myeloid leukemia (CML) [12] has allowed to elucidate the molecular dissection of chronic proliferation in MPN. Using CML as paradigm of constitutive activation of TK in chronic myeloproliferation, James et al. sequenced the coding exons and intron-exon junctions of *JAK2* in 3 polycythemia vera (PV) patients and 2 controls. In 2 of these patients a G-to-T mutation at nucleotide 1849 in exon 12 was found, leading to a substitution of valine to phenylalanine at position 617 (V617F). This mutation was not a polymorphism, but a recurrent acquired mutation that was found in granulocytes, erythroblasts, and platelets of 40 out of 45 PV patients but not in any controls or patients with secondary erythrocytosis [13]. *JAK2*V617F was also found in other BCR-ABL negative MPN [14]. *JAK2*V617F occurs in the pseudokinase domain of the *JAK2* gene. The mutated pseudokinase domain is not able to negatively regulate the kinase domain of *JAK2*, resulting in an autonomous activation of the *JAK2* kinase domain with subsequently persistent phosphorylation of STAT and MAPK proteins [15, 16] and hyperstimulation of the cytokine signalling pathway [17]. As a consequence, cells expressing the *JAK2*V617F mutation are hypersensitive to hematopoietic cytokine stimulation, resulting in an abnormal erythroid-, myeloid-, and thromboproliferation. Moreover, *JAK2*-deficient mice do not survive because of absence of erythropoiesis. Myeloid progenitors of these mice fail to respond to EPO, GM-CSF, and thrombopoietin stimulation [18]. These experiments demonstrate that *JAK2* plays an essential role in the development of normal hematopoiesis.

 Not all patients with “classical” MPN carry the *JAK2*V617F mutation. This mutation is present in almost all PV patients, but only in half of the patients with essential thrombocythemia (ET) and primary myelofibrosis (PMF), suggesting that there are alternative routes of *JAK*-cytokine signalling activation [14]. In the most prevalent *BCR-ABL1* negative MPN (PV, ET, and PMF), up to now it is known that *JAK2* activation may also occur by *JAK2* mutations in exon 12 [19], by mutations in the inhibitory adaptor protein LNK [20], or by a *TEL-JAK2* chromosomal translocation that induces erythropoietin-independent erythroid differentiation and myelofibrosis [21]. Other mutations described in MPN but also in other haematological malignancies are mutations in the thrombopoietin receptor at codon 515 (*MPL*) [22], the isocitrate dehydrogenase family genes (*IDH*), the additional sex combs like 1 gene (*ASXL1*), the casitas B-lineage lymphoma proto-oncogene (*CBL*), the TET oncogene family member 2 (*TET2*), and the Ikaros family zinc finger 1 gene (*IKZF1*) [23]. These mutations promote hematopoietic proliferation via other pathways than *JAK*-cytokine signalling activation and they have been detected more frequently in MPN other than PV, ET of PMF, myelodysplastic syndromes, secondary acute myeloid leukemia, and blast-transformation phase of MPN.

There is a relationship between the underlying genetic mechanism of the MPN and the response to different treatments. For example, 40–50% of the patients with primary PMF and ET who carried *JAK2*V617F mutation had decreases in proportion of *JAK2*-mutated DNA [24]. About 20% of the PMF and ET patients who carried *MPL* mutations had no decreases in proportion of *MPL*-mutated DNA when treated with *JAK2* inhibitors but they did have it when treated with biological response modifiers (pegylated-interferon alpha 2a, lenalidomide, and prednisone) [24].

 New biological properties of *JAK2* are currently being investigated. Recently, it has been demonstrated that *JAK2* is not only localised in the cytoplasm of hematopoietic cells where *JAK2* plays a role in the cytokine signalling pathway, but also in the nucleus of hematopoietic cells [25]. In the nucleus, a hyperactive *JAK2* induces phosphorylation of the histone H3 at tyrosine residue 41 (H3Y41), releasing the transcriptional repressor heterochromatin protein 1alfa (HP1a) from chromatin and resulting in an activation of transcription of genes repressed by HP1a, as the oncogene *LMO2*. *LMO2* might play a role in the pathogenicity of MPN. It is remarkable that H3Y41 phosphorylation requires a hyperactive *JAK2*, implying that the activation of different pathways with different *JAK2* effectors and different target genes is involved in the pathogenesis of MPN.

In the era of *JAK2* inhibitors, this finding opens new possibilities for combined therapeutic targets (*JAK2* inhibitors and histone posttranslational modificators) that may benefit patients with MPN [26].

## 2. Current Therapies for *BCR-ABL1* Negative MPN (PV, ET, and PMF) Based on *JAK2* Inhibitors and Their Combinations

Hyper-activation of *JAK2* is a critical step in the pathogenesis of *BCR-ABL1* negative, classical MPN.

Autonomous activation of the *JAK2* kinase domain with subsequently persisting phosphorylation of STAT and MAPK proteins occurs in patients with and without *JAK2*V617F mutations. *JAK2* inhibitors have been designed to suppress the cytokine signalling induced by a hyperactive cytoplasmatic *JAK2* gene. *JAK2* inhibitors compete for the ATP-binding pocket of the tyrosine kinase domain of *JAK2* [27]. Because *JAK2*V617F mutation is localized outside the ATP-binding site, *JAK2* inhibitors do not discriminate between *JAK2* and *JAK2*-mutated genes [28]. In consequence, *JAK2* inhibitors can be used in patients with MPN independently of the *JAK2* mutation status.

 Nowadays, several *JAK2* inhibitors are tested in clinical trials in Europe and USA (*INCB018424*, *TG101348*, *CYT387*, *CEP-701, AZD1480, SB1518, and XL019*) and others (*NCB016562*, *NVP-BSK805,* and *R723*) are in preclinical development.

*INCB018424* known as *Ruxolitinib* is a potent and selective *JAK1* and *JAK2* inhibitor. It has been used in patients with PMF, where *INCB018424* downregulates proinflammatory cytokines through the inhibition of *JAK1* and suppresses the phosphorylated signal of STAT3 through the inhibition of *JAK2*, independently of the presence of *JAK2*V617F mutation [29].

In animal models of *JAK2*V617F mutation MPN, oral *INCB018424* markedly reduced splenomegaly and circulating levels of inflammatory cytokines and preferentially eliminated neoplastic cells, resulting in significantly prolonged survival without myelosuppressive or immunosuppressive effects [30].

 In patients with PMF treated with *INCB018424*, a significant reduction of constitutional symptoms and more than 50% reduction of splenomegaly occurred. Clinical benefits were associated with a marked decrease of levels of circulating inflammatory cytokines, although the load of *JAK2*V617F was marginally reduced [29]. Myelosuppression grade 3 or 4 was observed in less than 10% of the patients.

*TG101348*, also known as *SAR302503*, is a selective small-molecule *JAK2* antagonist that inhibits primary hematopoietic cells derived from patients with MPN and with *JAK2*V617F, *MPL*W515K, *JAK2* exon 12 mutations as well as mutation negative patients [31]. In animal models of *JAK2*V617F positive MPN, *TG101348* reduced erythrocyte and leukocyte counts, the extramedullary hematopoiesis, and myelofibrosis without toxicities [31, 32]. Biologically, *TG101348* reduced the *JAK2*V617F disease burden, and it was demonstrated a suppression of endogenous erythroid colony formation and inhibition of phosphorylated STAT5 [31]. In patients with myelofibrosis, *TG101348* induced a reduction of the spleen size according to the criteria of International Working Group for Myelofibrosis Research and Treatment (IWG-MRT) [33] and a normalization of blood counts after 6 and 12 cycles [34]. A significant decrease in *JAK2*V617F allele burden was observed at 6 months in mutation-positive patients with a consistent decrease at 12 months [34].

*CYT387* is an ATP-competitive potent *JAK1*, *JAK2*, and tyrosine kinase 2 (*TYK2*) inhibitor, at nanomolar concentrations, more selective against *JAK2* than against other tyrosine kinases in comparison with other *JAK2* inhibitors [35]. Its effect has been analyzed in cell lines harboring mutated *JAK2* alleles, showing an inhibition of growth of erythroid colonies and erythroleukemia cells and inhibition of STAT5 phosphorylation.

In a murine MPN model, *CYT387* normalized erythrocytes, leukocytes, spleen size, and restored physiologic levels of inflammatory cytokines. Biologically, there was a reduction of the *JAK2*V617F allele burden. However, after cessing therapy, *JAK2*V617F positive cells persisted and MPN relapsed [35].

*CEP-701* known as *Lestaurtinib* is an Fms-like tyrosine kinase 3 (*FLT3*) inhibitor in current use on acute myeloid leukemia clinical trials and a *JAK2* kinase inhibitor which suppresses phosphorylation induced by *JAK2* tyrosine kinase. In patients with PV, *CEP-701* inhibited growth of expanded erythroid cells [36]. In 22 patients with PMF who carried out the *JAK2*V617F mutation, *CEP-701* induced a modest clinical recovery with mainly improvement of the spleen size. Biologically there was no improvement in bone marrow fibrosis or *JAK2*V617F allele burden [37]. Mainly toxicities have a high incidence of any grade of gastrointestinal toxicity in 9–72% of the patients and haematological toxicity grade 3-4 in 14–23% of the patients [37].

*JAK2* inhibitors can be compared with *BCR-ABL1* inhibitors, since both sort of drugs are TK inhibitors. Nevertheless, whereas *BCR-ABL1* inhibitors are directed against an aberrant fusion gene [38], *JAK2* inhibitors are directed against a gene which is present in normal cells and have an important role in the development of normal hematopoiesis. This means that adverse events are induced with *JAK2* inhibitors at doses to be able to control the myeloproliferative phenotype, inducing often grade 3-4 hematological toxicity as seen in clinical trials, limiting the clinical efficiency of *JAK2* inhibitors. Different studies describe the incidence of reversible grade 3 or 4 hematological toxicity between 3–35% depending on the inhibitor specificity [34, 37, 39]. Other common adverse events are gastrointestinal symptoms, probably related to the inhibition of other tyrosine kinases. The incidence of nausea, vomiting, and diarrhoea varies between 5–70% depending on the compound [34, 37, 39].

 Up to now it is known that *JAK2* is a member of a family of tyrosine kinases present in the cytoplasm of hematopoietic cells. Recently, it has been demonstrated that *JAK2* is also present in the nucleus of hematopoietic cells where it indirectly activates the expression of oncogenes as *LMO2* [25]. It is not yet well known whether *JAK2* inhibitors have a role in the inhibition of the *JAK2* nuclear function. In the next years, the increasing clinical and biological experience with *JAK2* inhibitors will clarify their role.

Although imatinib treatment in CML cannot directly be compared with *JAK2* inhibition in MPN, it can be used as a model of clinical experience with TK inhibitors. Therefore, we can speculate about what it is going to happen with the use of *JAK2* inhibitors in the clinical practice. One might expect the drug resistance to *JAK2* inhibitors by acquisition of mutations in the ATP-binding pocket of the TK domain of *JAK2* and/or via the amplification of *JAK2*. We can also expect that *JAK2* inhibitors are efficient to alleviate the clinical symptoms of patients with MPN, when used as monotherapy, but inefficient to cure the disease as happened with imatinib [40]. Clinical trials using *JAK2* inhibitors for patients with myelofibrosis have demonstrated the effectiveness of these drugs to reduce clinical symptoms splenomegaly and to improve quality of life. However, in these trials, *JAK2* burden was slightly reduced, indicating that *JAK2* inhibitors are efficient to block the cytokine pathway responsible of the clinical symptoms in patients with MPN, but are not enough efficient to block the main molecular mechanism that originates the disease. Recently, Mullally et al. [41], using a *JAK2*V617F knock-in mouse MPN model, have shown that *JAK2* inhibitors are able to control the hyperproliferation of committed hematopoietic progenitors in MPN, but are unable to eliminate the cell population from which the initiator clone arises. This cell population has been identified as noncommitted hematopoietic progenitors *JAK2*V617F+, Lin−, Sca+ c-kit+ (LSK positive). *JAK2* inhibitors may have to be used in combination with drugs which target the LSK positive noncommitted population to be able to cure MPN.

 Given the fact that *JAK2* inhibitors induce myelosuppression but cannot cure MPN, combinations with other compounds that might have therapeutic synergy with *JAK2* inhibitors seem to be mandatory. In this sense, interferon-alpha therapy is a good option to be associated to *JAK2* inhibitors, due to its multiple effects on the regulation of immune modulatory cells, the expression of apoptotic genes, inhibition of angiogenesis, suppression of the proliferation of hematopoietic progenitor cells, and promoting the cycling of hematopoietic stem cells [42, 43]. It is thought that interferon-alpha can also inhibit the cytokine signalling coming from bone marrow stromal cells to support proliferation and survival of malignant cells in MPN. Recently, Manshouri et al. have demonstrated that humoral factors secreted by the bone marrow stromal cells protect malignant cells carrying *JAK2*V617F from the therapeutic effect of the *JAk2* inhibitors [44]. Thus, combination of *JAK2* inhibitors and interferon alpha could be a more efficient therapeutic regimen to treat MPN patients than only *JAK2* inhibitors.

 Other immunomodulatory drugs are also been tested in MPN patients, mainly in those with myelofibrosis. Thalidomide and lenalidomide with or without prednisone have shown efficacy to inhibit the increased cytokine production in these patients, decreasing the spleen size, myelofibrosis, and inhibiting angiogenesis [45]. Pomalidomide, another analogue, is currently being evaluated with or without prednisone in large clinical trials to treat patients with myelofibrosis [46]. These immunomodulatory drugs are candidates to be associated to *JAK2* inhibitors as targeting therapy in patients with MPN.

 Classical therapies, as hydroxycarbamide, are also effective to treat patients with MPN, not only as cytoreduction therapy but also as therapy to decrease the *JAK2*V617F load. Recently, Besses et al. have shown that hydroxycarbamide can reduce the *JAK2* mutant load to more than 50% in untreated patients diagnosed with PV and TE [47]. This effect has synergy with the therapeutic effect of *JAK2* inhibitors, making hydroxycarbamide a candidate therapy to be combined with *JAK2* inhibitors.

*JAK2* inhibitors are effective to alleviate clinical symptoms in patients with *BCR-ABL* negative MPN. Combination with other therapies which show synergy and other biological properties than *JAK2* inhibitors is promising as the most effective therapy in these disorders (Table 1).

## 3. Final Remarks

*JAK2* is a tyrosine kinase gene which plays an essential role in the development of normal haematopoiesis and in the cytokine signalling pathway involving STAT3 and STAT5 proteins. Hyperactivation of *JAK2* occurs in MPN by different genetic mechanisms. *JAK2* inhibitors have been designed to suppress the cytokine cascade initiated by the activation of *JAK2*, independently of the underlying genetic mechanism. In phase II/III of clinical trials, *JAK2* inhibitors are efficient to control clinical symptoms and to improve quality of life. Recently, it has been demonstrated that *JAK2* can also be located in the nucleus of cells, where it plays a role in the transcriptional control of genes regulated by HP1a. Whether *JAK2* inhibitors may have a role in the suppression of the nuclear role of *JAK2* or not is at the moment unknown, but it opens new insights for the combined use of epigenetic therapy with *JAK2* inhibitors for the treatment of MPN.

 Recently, it has also been demonstrated that *JAK2* inhibitors are unable to target uncommitted hematopoietic progenitors responsible of the initiation of the myeloproliferative disease. It is expected that, in order to cure the MPN disease, *JAK2* inhibitors should be combined with other drugs to target simultaneously different pathways and to target the initiator hematopoietic LSK positive cell population in MPN.

 Taking advantage of the inhibition of the cytokine cascade of *JAK2* inhibitors, these compounds are going to be used not only to treat patients with MPN, but also patients with autoimmune diseases as rheumatoid arthritis, Crohn's disease, or Colitis ulcerosa.

## Figures and Tables

**Table 1:** Current therapies for *BCR-ABL1* negative MPN patients to be combined with *JAK2* inhibitors.

Drug	Target	Results	*JAK2*V617F load	Reference
*JAK2* inhibitors				
INCB018424 Ruxolitinib	*JAK2*, *JAK1 *	>50% reduction in splenomegaly and constitutional symptoms	*JAK2*V617F load marginally reduced	Verstovsek et al. [29]
TG101348 or SAR302503	*JAK2*, *FLT3 *	Reduction in splenomegaly	*JAK2*V617F load significantly reduced	Pardanani et al. [34]
*CYT387 *	*JAK2*, *JAK1*, *TYK2 *	In a murine model, normalized erythrocytes, leukocytes, spleen size, and levels of inflammatory cytokines	*JAK2*V617F load reduced	Tyner et al. [35]
*CEP-701 * *Lestaurtinib *	*JAK2*, *FLT3 *	Reduction in splenomegaly	*JAK2*V617F load no reduced	Hexner et al. [36] Santos et al. [37]

Immunomodulators				
Interferon-alpha	Stromal cells	Inhibit cytokine signalling, proliferation and angiogenesis	Unknown	Kiladjian et al. [43]
Thalidomide	Stromal cells	Inhibition of angiogenesis	Unknown	Jabbour et al. [45]
Lenalidomide	Stromal cells	Inhibition of angiogenesis	Unknown	Jabbour et al. [45]
Pomalidomide	Stromal cells	Inhibition of angiogenesis	Unknown	Begna et al. [46]

Other				
Hydroxycarbamide	*JAK2* mutant burden	Inhibition of proliferation	*JAK2*V617F load significantly reduced	Besses et al. [47]
Histone post-translationa modificators	*JAK2* nuclear	Depletion of *JAK2*V617F autophosphorylation, apoptosis in vitro	Unknown	Wang et al. [26]
